# Potentially Pathogenic *Leptospira* in the Environment of an Elephant Camp in Thailand

**DOI:** 10.3390/tropicalmed5040183

**Published:** 2020-12-06

**Authors:** Somjit Chaiwattanarungruengpaisan, Wasinee Thepapichaikul, Weena Paungpin, Kanokwan Ketchim, Sarin Suwanpakdee, Metawee Thongdee

**Affiliations:** 1 The Monitoring and Surveillance Center for Zoonotic Diseases in Wildlife and Exotic Animals, Faculty of Veterinary Science, Mahidol University, Salaya, Nakhon Pathom 73170, Thailand; somjit.cha@mahidol.ac.th (S.C.); weena.pau@mahidol.ac.th (W.P.); kanokwan.ket@mahidol.ac.th (K.K.); sarin.suw@mahidol.ac.th (S.S.); 2 Livestock and Wildlife Hospital, Faculty of Veterinary Science, Mahidol University, Sai Yok, Kanchanaburi 71150, Thailand; wasinee.the@mahidol.ac.th; 3 Department of Clinical Sciences and Public Health, Faculty of Veterinary Science, Mahidol University, Salaya, Nakhon Pathom 73170, Thailand

**Keywords:** *Leptospira*, environment, elephant camp, isolation, recreational activities

## Abstract

*Leptospira* is the causative agent of leptospirosis, a globally emerging zoonotic disease. The infection is commonly acquired through contact with the contaminated environment. To extend the knowledge on environmental source of leptospirosis, we investigated the presence of *Leptospira* in an elephant camp setting where the interaction between humans, animals, and the shared environment occur particularly when engaging in recreational activities. In this study, a total of 24 environmental samples were collected from an elephant camp area in western Thailand. All samples were processed for *Leptospira* isolation using the EMJH medium. The identification of *Leptospira* species was carried out by partial 16S rRNA and *secY* gene sequencing. Of those 24 samples, 18 samples (75%) were culture-positive for *Leptospira*. The recovered leptospires were mostly derived from water and soil sampled from a river and a mud pond, the main areas for recreational activities. The majority of the isolates were classified into “Pathogens” clade (89%, 16/18) and more than half of the isolates (61%, 11/18) contained species of the “Saprophytes” clade. Notably, two soil isolates from the river beach sampling area were found to contain leptospiral DNA with high similarity to the pathogenic *L. interrogans* and *L. santarosai*. The evidence of diverse *Leptospira* species, particularly those belonging to the “Pathogens” clade, suggest that the shared environments of an elephant camp can serve as potential infection source and may pose a risk to the elephant camp tourists and workers.

## 1. Introduction

Leptospirosis is a worldwide distributed zoonotic disease caused by spirochete bacteria in the genus of *Leptospira* [1]. Currently, there are 64 named species belonging to the genus *Leptospira* in which more than half were originally isolated from the environment [2]. Based on comparative genomic analyses, the recent classification has divided *Leptospira* into two major clades, including Pathogens (P) and Saprophytes (S); each clade has been further subdivided into two subclades. Pathogen subclades are composed of P1 (formerly known as a pathogenic group) and P2 (formerly known as an intermediate group), while Saprophytes subclades consist of S1 (formerly known as a saprophytic group) and S2 (a new subclade) [2]. Pathogenic leptospires have a complex life cycle. The bacteria colonize the renal tubules of chronically infected asymptomatic animal reservoirs and are excreted through urine into the environment [1,3]. The human exposure route can be either direct contact with the urine of infected animals or indirect contact with the contaminated environment [1,3,4].

Leptospirosis has been increasingly recognized as an environmental-borne infection [5]. A great number of cases of leptospirosis occur after engaging in both occupational and recreational activities in which people can become infected through the contact between wounded skins or mucous membranes and contaminated water or soil [6,7]. Indirect environmental exposure accounts for several occupational activities such as planting, farming, sewer maintenance, military activities, etc. [5,8,9]. In recent years, the growing incidence of leptospirosis has been associated with recreational and water-based activities [10,11]. Moreover, some outbreaks of leptospirosis have been linked to the recreational exposure, for example, an outbreak associated with swimming in the creek in Malaysia [12], and outbreaks associated with freshwater sports in France [13] and Switzerland [14]. 

Numerous studies attempted to investigate the presence and persistence of *Leptospira* in the environment to better understand the epidemiology of leptospirosis. It is assumed that pathogenic *Leptospira* could not multiply in the environment [15]. However, other studies have confirmed the pathogen’s ability to survive and remain virulent for several weeks in water and soil, in which the survival ability may rely on the species and strains [15,16,17,18,19]. Although the environment is not a multiplication reservoir of pathogenic leptospires, it can serve as a temporary carrier of the pathogen, which can considerably enable the transmission of the disease [15,16,20].

The role of the environment in leptospirosis epidemiology is important. Several relevant aspects are needed to be fully elucidated such as the environmental factors driving the survival of leptospires, environmental ecology, and survival mechanism of *Leptospira* in the environment [21]. However, the crucial knowledge gap remains concerning the understanding of the survival and persistence of virulent *Leptospira* in the environment [21]. Although a variety of environmental settings has been investigated for the presence of *Leptospira*, several natural settings related to occupational and recreational activities remain underexplored. An elephant camp is one of the underexplored settings. Recreational activities in the elephant camp such as elephant riding and bathing are strongly associated with environmental exposure. Tourists and elephant camp workers are at risk of leptospirosis infection through activity participation. To date, no human cases of leptospirosis have been linked to the recreational activities in the elephant camp. However, the environment of this setting can possibly act as a potential source of infection.

In the current study, we investigated the presence of *Leptospira* in an elephant camp setting where the interaction between humans, animals, and the shared environment occurs particularly when engaging in recreational activities. The knowledge of the *Leptospira* habitat and distribution in the water and soil environment can help to determine the environmental risk of leptospirosis and allow the implementation of appropriate prevention and control measures.

## 2. Materials and Methods 

### 2.1. Location and Description of the Study Site

This study was conducted in February 2018 in an elephant camp in western Thailand. The elephant camp that served as the location for this study is a natural jungle setting located along a river where elephants riding and bathing takes place regularly. Sampling sites were chosen to represent environmental areas of the setting which frequently share between humans and elephants including a river, a mud pond, and an elephant barn. 

### 2.2. Environmental Sample Collection 

**Figure 1:**
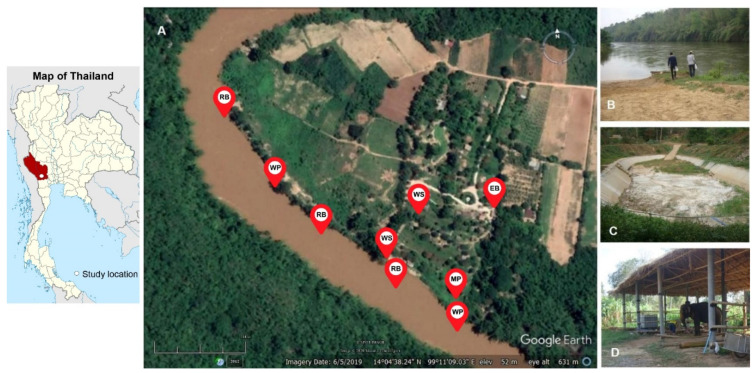
Map of the study location and the sampling locations of the environmental samples in the elephant camp areas (**A**). The sampling spots were marked on the map as follows: EB; Elephant barn, MP; Mud pond, RB; River beach, WP; Water pumping station and WS; Water supplies. The images of the sampling locations were shown on the right panel including a river beach (**B**), a mud pond (**C**), and an elephant barn (**D**).

Water and soil samples were collected from each of the sampling sites in the elephant camp as illustrated in Figure 1. The main sampling area was undertaken along the river in the range of 0.64 kilometres, where three river beaches and two water pumping stations were considered to be the location of sampling. Soil samples were collected at the river coast and the shoreline, while water samples were collected at the shoreline. Another sampling area in the elephant camp was a mud pond where the elephants normally take a mud bath with tourists. The pond is a long rectangular man-made pond with a depth of approximately 3 m. During the survey period, the pond was temporarily closed, allowing for the collection of muddy soil and stagnant water at the bottom of the pond. Additional soil samples were collected from the area of an elephant barn where the elephants usually stay during the daytime. Moreover, water sampling was carried out from non-drinking water supplies in the elephant camp.

One hundred mL of each water sample was collected in sterile tubes; 200−300 g of each soil sample was picked up in a sterile bag. The temperature and pH of the water were measured at each sampling point while the measurement of those parameters in soil was not conducted in this study. The geographical position of each sampling point was recorded to create a map showing the location of each sampling point by using the Google Earth program version 7.1.8.3036 (Google, Mountain View, CA, USA). All samples were kept in an opaque container and transported under the ambient condition to the laboratory. 

### 2.3. Environmental Sample Processing for Leptospira Isolation

All samples were processed for *Leptospira* isolation according to the earlier study [22]. Briefly, for the water sample, approximately 30 mL of each water sample was passed through a 0.2 µm filter. A 0.5 mL of the filtrate was inoculated, in duplicate, into 2 mL of the liquid EMJH medium containing 100 µg/mL of 5-fluorouracil (5-FU). The rest of the filtrate was centrifuged at 2200× *g* for 20 min. The pellet was inoculated into 2 mL of the semi-solid EMJH medium containing 0.2% of Noble agar base and 100 µg/mL of 5-FU. As for soil samples, approximately 50 g of each soil sample was added with 100 mL phosphate buffered saline to make soil suspension and was manually mixed before leaving to sediment for 30 min at room temperature. Afterward, watery part was centrifuged at 250× *g* for 10 min to precipitate large debris before passing through a 0.2 µm filter. The filtrate was inoculated into the liquid and semi-solid EMJH medium as mentioned above. The cultures were maintained at 28 °C and were examined the presence of *Leptospira*-like microorganism under a dark field microscope weekly. A negative result was considered only when *Leptospira*-like microorganism was not detected from the culture within two months after sample inoculation.

### 2.4. Genomic DNA Extraction

Bacterial genomic DNA was extracted from a confluent liquid culture using the Genomic DNA Mini Kit (blood and cultured cell) (Geneaid, New Taipei City, Taiwan). One mL of a confluent liquid culture was harvested by the centrifugation at 20,000× *g* for 10 min; the pellet was subsequently taken for genomic DNA extraction according to the manufacturer’s instruction. The extracted DNA was finally suspended in 30 µL of Tris-EDTA buffer and stored at −20 °C until assay.

### 2.5. PCR Amplification of 16S rRNA and secY Genes

An amplification of the partial 16S rRNA gene, using primers specific for pathogenic and intermediate groups of *Leptospira*, was carried out following a published method [23] with some modifications. Instead of a nested PCR assay with four primers, we performed a single-round conventional PCR using only the two outer primers. The primers used were as follows: rrs-outer-F (5′CTCAGAACTAACGCTGGCGGCGCG3′) and rrs-outer-R (5′GGTTCGTTACTGAGGGTTAAAACCCCC3′). A total volume of 25 µL PCR reaction contained 1X MgCl_2_ free PCR buffer, 2.5 mM of MgCl_2_, 200 µM of dNTP, 1.25 unit of Taq DNA polymerase (iNtRON Biotechnology Inc, Gyeonggi-do, Korea), 0.5 µM of each outer primer, 1 M of Betaine (Sigma-Aldrich, St. Louis, MO, USA), and 2 µL of DNA extracted from *Leptospira* culture. PCR was performed in a PCT-200 Peltier Thermal Cycler (MJ Research, Inc., Waltham, MA, USA) using the following conditions: one cycle of initial denaturation at 95 °C for 2 min, 40 cycles of denaturation at 95 °C for 10 s, annealing at 67 °C for 15 s, and elongation at 72 °C for 30 s; followed by one cycle of final extension at 72 °C for 7 min. Positive and negative controls were included in each run. The PCR product of 810 bp size was analyzed by 1.5% agarose gel electrophoresis.

DNA samples showing no PCR product in the first PCR were subsequently examined in the second PCR using 16S rRNA primers specific for the *Leptospira* genus as described earlier [24]. The primers were as follows: rrs-F (5′GTTTGATCCTGGCTCAG3′) and rrs-R (5′CCGCACCTTCCGATAC3′). PCR reactions were prepared in a volume of 25 µL containing all components as mentioned above, except 1 M of Betaine. PCR was performed in a PCT-200 Peltier Thermal Cycler (MJ Research, Inc., Waltham, MA, USA) using the following conditions: one cycle of initial denaturation at 94 °C for 2 min; 30 cycles of denaturation at 94 °C for 1 min, annealing at 55 °C for 45 s, and elongation at 72 °C for 1.5 min; followed by one cycle of final extension at 72 °C for 10 min. Positive and negative control of each run were included. The amplified product of 1483 bp size was analyzed by 1.5% agarose gel electrophoresis.

For amplification of the *secY* gene of *Leptospira* spp., the primers G1 (5′CTGAATCGCTGTATAAAAGT3′) and G2 (5′GGAAAACAAATGGTCGGAAG3′) were used as previously described with some modifications [25]. A 25 ul PCR reaction was prepared for each DNA sample as earlier mentioned. PCR was conducted in a PCT-200 Peltier Thermal Cycler (MJ Research, Inc., Waltham, MA, USA) using the following conditions: one cycle of initial denaturation at 94 °C for 2 min; 35 cycles of denaturation at 94 °C for 20 s, annealing at 55 °C for 15 s, and elongation at 72 °C for 30 s; followed by one cycle of final extension at 72 °C for 5 min. Positive and negative controls of each run were included. The amplified product of 285 bp size was analyzed by 1.5% agarose gel electrophoresis.

### 2.6. DNA Sequencing and Phylogenetic Analysis

The PCR products with predicted size were purified from agarose gel using GenepHlow^TM^ Gel/PCR Kit (Geneaid, New Taipei City, Taiwan) according to the manufacturer’s instruction. The purified PCR products were sequenced by Macrogen (Seoul, Korea) and the trimmed nucleotide sequences were deposited in the GenBank database. Nucleotide sequence analysis was carried out using the NCBI BLAST (https://blast.ncbi.nlm.nih.gov/Blast.cgi). A phylogenetic tree was constructed based on partial nucleotide sequences of the 16S rRNA and *secY* genes by the Neighbor-Joining method with the Kimura 2-parameter model and the Tajima-Nei model, respectively, in the MEGA program version 7.0.

## 3. Results

A total of 24 samples were collected from the environmental areas of the elephant camp (n = 24: water = 12, soil = 12). More than half of the environmental samples were collected from the river (n = 15), and the remaining samples were collected from the mud pond (n = 3), the elephant barn (n = 3), and the water supplies in the elephant camp (n = 3). During the sampling period, the temperature of the water samples ranged from 27 °C to 34 °C, and the pH ranged from 8.8 to 9.2.

The successful isolation of *Leptospira* spp. was 75% (n = 18/24) with 58% (n = 14/24) and 71% (n = 17/24) culture yield in the liquid and semi-solid EMJH medium, respectively. The recovery percentage of leptospires from water and soil samples was 83% (n = 10/12) and 67% (n = 8/12), respectively. Positive cultures for *Leptospira* were found in most samples from the river (n = 12/15, 80%) and all samples from the mud pond (n = 3/3, 100%) and the water supplies in the elephant camp (n = 3/3, 100%). None of the soil samples collected from the elephant barn (n = 0/3) were positive for *Leptospira* isolation.

**Figure 2:**
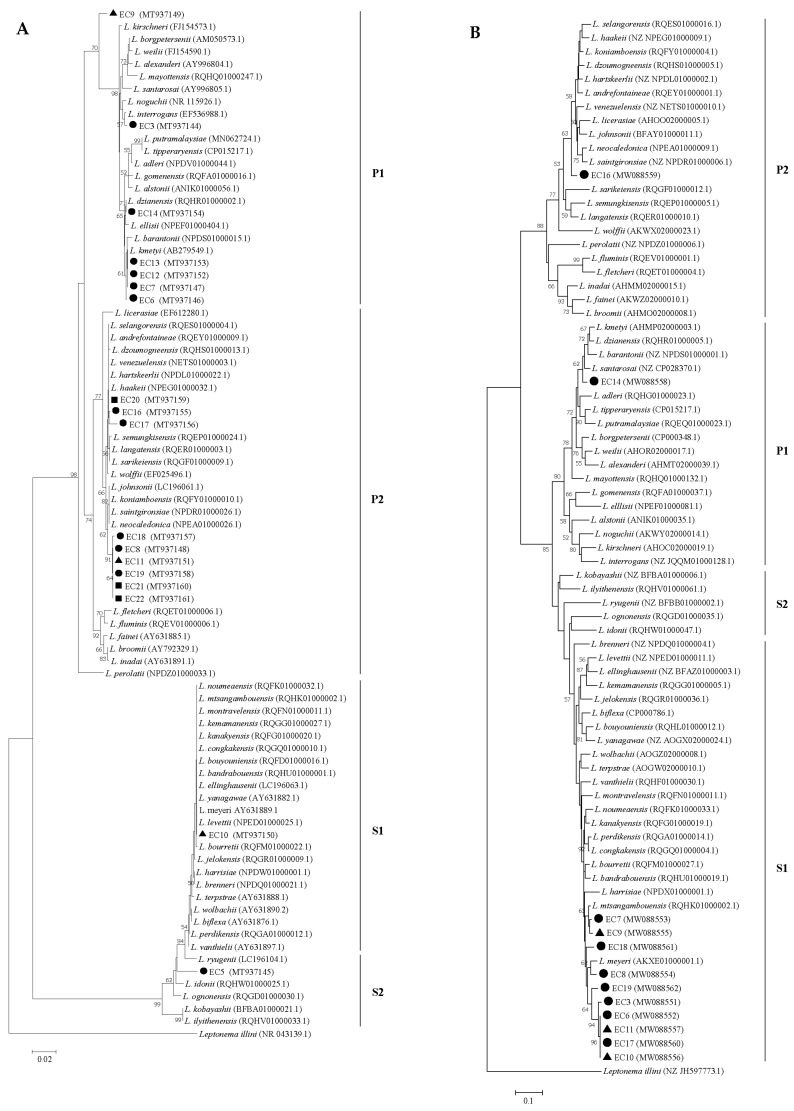
Neighbor-joining phylogenetic tree of partial 16S rRNA (**A**) and *secY* (**B**) sequences of *Leptospira* isolated from the environmental samples of the elephant camp areas. Circle, triangle, and square indicated the environmental isolates from a river, a mud pond, and water supplies, respectively. A bootstrap of 1000 replicates was performed and values above 50% are shown in the nodes. *Leptonema illini* was used as the outgroup. The GenBank accession numbers of our isolates and reference species are shown in the parentheses.

The 16S rRNA PCRs demonstrated that the majority of culture-positive samples (89%, n = 16) contained either pathogenic or intermediate *Leptospira*, whereas the remaining samples (11%, n = 2) consisted of only non-pathogenic ones (Table S1). BLASTn analysis of 16s rRNA sequences amplified from each environmental isolate is shown in Table S2. The phylogenetic tree analysis based on partial 16S rRNA gene sequences of our 18 environmental isolates (GenBank accession numbers MT937144-MT937161) showed that the isolates were categorized into two major clades of *Leptospira* spp.; Pathogens (P) and Saprophytes (S) (Figure 2A) according to the new classification [2]. Seven isolates belonged to the subclade P1 on the branch related to *L. interrogans* (n = 1), *L. dzianensis* (n = 1), and *L. kmetyi* (n = 4), where one isolate was separated into a different branch. The other nine isolates were clustered within the subclade P2 on the branch related to *L. haakeii* (n = 3) and *L. neocaledonica* (n = 6). The remaining two isolates resided on a branch of *L. levettii*, the member of S1 subclade and *L. idonii*, the member of the S2 subclade.

On the other hand, the *secY* PCR was positive in 12 out of 18 culture-positive samples (66.6%) (Table S1). BLASTn analysis of *secY* sequences amplified from each environmental isolate demonstrated that two out of 12 isolates were close to *L. santarosai* (n = 2; 96 and 77% identity), while the remaining isolates were close to *L. biflexa* (n = 10; 88−91% identity) (Table S2). The phylogenetic tree analysis based on partial *secY* gene sequences of 12 environmental isolates (GenBank accession numbers MW088551-MW088562) showed that the isolates were separated into the Pathogens and Saprophytes clades (Figure 2B). Within the Pathogen clade, one isolate was positioned in a branch related to *L. santarosai* and the other one was located on the branch related to *L. saintgironsiae*. The remaining 10 isolates were classified within the lineage of saprophytes. Of those, seven isolates were grouped on the branch related to *L. meyeri* and three isolates were grouped on the branch related to *L. mtsangambouensis*.

The number and percentage of *Leptospira* spp. recovered from the water and soil samples of each sampling location in the elephant camp were shown in Table 1. Overall, the large proportion of the species in the Pathogens clades (66.6%, n = 16/24) was present in the environmental samples of the elephant camp sampling areas while the species in the Saprophytes clades were present in the smaller proportion (45.8%, n = 11/24). The mix of the species belonging to the Saprophytes and Pathogens clades in the same environmental samples could be determined in 37.5% (n = 9/24) of the environmental samples based on 16S rRNA and *secY* PCR and gene sequencing. *Leptospira* species in subclade P1 and P2 were isolated from the majority of the environmental samples derived from the river in which the positive water and soil samples were 63% (n = 5/8) and 86% (n = 6/7), respectively. Notably, the partial 16S rRNA and *secY* nucleotide sequences with high similarity to the pathogenic *L. interrogans* and *L. santarosai* could be detected in soil samples from the river beach sampling area. Leptospires from the P1 and P2 subclades were also recovered from the water sample (100%, n = 1/1) and the soil sample (50%, n = 1/2) were collected from the mud pond. Moreover, all of the sampling water (100%, n = 3/3) from the water supplies in the elephant camp were found to contain *Leptospira* subclade P2 species. On the other hand, 11 environmental samples were found to harbor saprophytic species. Ten out of 11 samples contained species of the subclade S1, of which 9 samples (7 river samples and 2 mud pond samples) were found to be mixed with species of the Pathogens clade. Only one water sample from the river contained *Leptospira* subclade S2 species.

## 4. Discussion

The current study reveals the presence of *Leptospira* spp. in the environmental areas of an elephant camp in western Thailand. Up to 75% (18/24) of the sampled environment in this setting contained leptospires. The viable *Leptospira* was predominantly found in water and soil derived from a river and a mud pond where the recreational activities such as elephant riding and bathing frequently take place. Additionally, leptospires could be obtained from all samples of water supplies in the camp. The water was originally pumped from the river and distributed around the camp to use for gardening and household activities. Conversely, soil samples collected from the area of the elephant barn were found to be free from viable *Leptospira*. The soil in this sampling area was noticeably hard and dry which was likely unfavorable for *Leptospira* survival [17]. It is the fact that several environmental factors such as pH, temperature, salinity, and moisture have been influenced the survival of *Leptospira* [1,26]. The pH (<6 to >8) and temperature (4 °C to 37 °C) allowing leptospires to survive have been earlier demonstrated from either field settings or laboratory settings [17,18,19,26,27,28,29] Accordingly, the pH (8.8 to 9.2) and temperature (27 °C to 34 °C) of the water samples observed in this study could support the survival of leptospires although the pH of the water was slightly more alkaline than the previous reports. While the pH and temperature data of the soil samples were not available in this study.

A substantial number of *Leptospira* species belonging to the Pathogens clade were successfully isolated from the environmental samples of the elephant camp. Most of the isolated strains were closely related to the novel species in P1 and P2 subclades which the virulence status has not been proven [2,16,20]. This finding was similar to other studies in which isolated strains recovered from the environmental samples were not responsible for leptospirosis infection in humans and animals [2,17,20,30,31,32,33,34]. Nevertheless, one soil isolate from the river beach sampling area was found to be closely related to the pathogenic *L. interrogans*, the species responsible for the human and animal infection. However, we failed to further characterize the soil isolate using Multi Locus Sequence Typing (MLST). The reason that may account for this failure was possibly due to the limitation of the number of pathogens presented in the crude culture. Besides, the pathogens could not be recovered even though we attempted to perform single colony isolation on solid agar. Consistent with our finding, the previous study has demonstrated that the *L. interrogans* strain was difficult to isolate from the environmental sample and the initial bacterial load could affect the success of bacterial isolation [33]. In their experiment, at least 10^6^ colonies/mL of the initial bacterial load is required for the isolation of *L. interrogans* strain from the water samples [33]. Furthermore, the abundance of the fast-growing saprophyte species in the environment is a major concern to hinder the isolation of the fastidious, slow-growing pathogenic species especially the virulent strains from water and soil in natural settings [22,31,34]. Only few studies have reported the successful isolation of the virulent pathogenic leptospires from the environmental samples, in which a traditional isolation method using animal inoculation had been employed in the past [35,36,37,38,39].

The other one soil isolate from the river beach sampling area was found to be related to species of the subclade P1. The possible species could be either *L. dzianensis* identified by 16S rRNA or *L. santarosai* identified by *secY* gene sequence analysis. *L. dzianensis* was originally isolated from a water sample in Dziani, a lake in Mayotte [2]. This novel species has not been proven for their virulence [2]. On the other hand, *L. santarosai* is a known causative agent of human leptospirosis in many countries worldwide such as Taiwan, Columbia, Costa Rica, the French West Indies, and the Peruvian Amazon [40,41,42,43,44]. This species has been isolated from various animal hosts such as dog, rats, marsupial, buffaloes, and cattle [45,46,47,48]. Conversely, there was little evidence of the presence of *L. santarosai* in environmental settings. Previous study of molecular analysis of environmental surface water in the Peruvian Amazon showed the presence of *Leptospiral* DNA sequences similar to *L. santarosai* in rural environmental water sources [44]. The findings suggested an environmental risk for leptospirosis exposure in the Peruvian Amazon, which is consistent with the previous isolation of pathogenic *L. santarosai* from leptospirosis patients and livestock (including cattle and pigs) in the same studied rural areas [44].

Moreover, the other species of subclade P1, *L. kmetyi*, was recovered from water and soil samples of the river beach sampling area. Although *L. kmetyi* has been distributed in a wide variety of environments [30,49,50,51], clear evidences of the species to cause diseases in humans and animals are scanty. The previous report identified *L. kmetyi* DNA in the blood sample of a leptospirosis-suspected patient who participated in canyoning activities on the Caribbean island of Martinique [43,52]. In addition, *L. kmetyi* was found to be a predominant species isolated from soils around leptospirosis patients’ houses in Kelantan, Malaysia [30]. Recently, the investigation of the source of leptospirosis outbreak among reserve military recruits in the Hulu Perdik forest, Malaysia, revealed that the pathogenic strains of *L. kmetyi* could be isolated from the environmental samples of the campsite outbreak area [51]. However, there was no relationship between the causative agents identified from the patients and the pathogenic strains recovered from the relevant environment [51]. Thus, the possible role of *L. kmetyi* in human infection still need to be elucidated.

Furthermore, three species of the subclade P2 including *L. haakeii*, *L. neocaledonica,* and *L. saintgironsiae* could be isolated from the environmental samples derived from the river, the mud pond, and the water supplies of the elephant camp. These newly described species were originally isolated from soil samples in New Caledonia [20]. The experiment of animal infection demonstrated that all of these novel species were unable to establish either acute infection or renal colonization [16].

Regarding the saprophytic species, it should be noted that the presence of saprophytes in the environmental samples of the elephant camp areas were not completely investigated since we did not conduct the genus-specific 16S rRNA PCR amplification in all culture-positive samples. Therefore, the presence of saprophytes in the samples cannot be ruled out, particularly in the culture-positive samples that were primarily confirmed by PCR to contain P1 and P2 subclades species. However, saprophytic species could be additionally identified in the environmental samples by *secY* PCR and gene sequencing. Moreover, the mix of the species belonging to the Saprophytes and Pathogens clades in the individual environmental samples could be observed in our study when we combined the results of 16s rRNA and *secY* gene sequence analysis. The environmental isolates close to species of subclade S1 (*L. levettii, L. meyeri* and *L. mtsangambouensis*) and the species of subclade S2 (*L. idonii*) could be obtained from the river and the mud pond sampling areas. Those species are not responsible for human and animal infection [2].

Elephants have not been recognized and received attention in term of being the animal reservoir host of pathogenic *Leptospira*. However, the recent study has reported the detection of *L. interrogans* in the urine of the domesticated elephants in Sri Lanka [53]. Four out of 13 elephants (31%) were confirmed to shed pathogenic leptospires in their urine by using *flaB* specific PCR detection and DNA sequencing of *flaB* gene revealed that all positive elephants were infected with *L. interrogans*. This study demonstrated that elephants can serve as the carrier of pathogenic leptospires and pose a high risk of contaminating the environment [53]. In addition, the captive elephants in India showed antibody titers against two serovars of *L. interrogans* including Australis and Canicola [54]. Likewise, Asian elephants in the northern and western parts of Thailand were found to have antibody titers against *L. interrogans* serovars Sejroe, Tarassovi, Ranarum, Bataviae, and Shermani [55]. The detection of antibodies against pathogenic leptospires in elephants indicated the possibility of leptospiral infection in the elephants. Although all elephants living in our studied area had no detectable leptospiral DNA in the urine by routine PCR in our laboratory (unpublished data), the evidence of *Leptospira* presence especially virulent pathogenic strains in the environment of the elephant camp should be taken into consideration.

There are several limitations in our study that should be addressed. Firstly, the study lacked the characterization of *Leptospira* species from the axenic cultures of the environmental samples. Multiple species of *Leptospira* can be present in individual environmental samples (water and soil) as demonstrated by previous studies [17,20]. The axenic culture would allow better identification of *Leptospira* species in the environmental sample and also provide the information of the *Leptospira* populations in the investigated environment. Secondly, the molecular characterization by *secY* PCR assay had limitations to detect some *Leptospira* species. The set of *secY* primers G1 and G2 used in this study cannot amplify *secY* gene fragment from pathogenic *L. kirschneri* and also several saprophytic species of *Leptospira* [25]. In our study, these primers could not amplify *secY* fragment of *L. kmetyi* (P1 subclade), *L. haakeii* and *L. neocaledonica* (P2 subclade), and *L. idonii* (S2 subclade) from the crude culture samples. Other *secY* primer sets have failed to amplify DNA fragments of *L. kmetyi* in blood samples of acute patients [43]. Nevertheless, the analysis of the G1-G2 region of the *secY* gene showed that this region provided sufficient information for *Leptospira* spp. discrimination [56]. Moreover, the phylogenetic tree based on the G1-G2 segment are in accordance with the 16S rRNA-based tree, indicating that analysis of this small fragment can be used to identify *Leptospira* species [56]. However, the use of *secY* primers for characterization of *Leptospira* in the environmental samples need more careful consideration. Thirdly, our study lacked data regarding the distribution of *Leptospira* spp. in human and animal populations in the same studied area. The information on epidemiological status in the relevant sectors including humans, animals, and environment in the same locality could help to better understand disease transmission and help to develop practical strategies for improving prevention and control of leptospirosis. Lastly, our study was conducted with a small environmental sample size that may not be representative of the entire population. However, a significant proportion of pathogenic *Leptospira* recovered from the environment of the elephant camp area implies the high risk of contracting leptospirosis and highlights the need for more awareness among elephant camp tourists and workers when engaging in outdoor activities related to environmental exposure. Moreover, our findings may help inform the appropriate prevention and control measures aimed at minimizing the exposure of humans to contaminated environments in this particular setting and also other similar settings.

## 5. Conclusions

This study revealed the evidence of *Leptospira* presence in the water and soil environment of an elephant camp setting, notably in the river and the mud pond, which are the main areas for recreational activities. The high prevalence of potentially pathogenic *Leptospira* spp. found in the environmental areas of an elephant camp should be taken into consideration as potential sources of leptospirosis infection. Our findings highlight the need for raising awareness of leptospirosis among the elephant camp tourists and workers. Appropriate prevention and control measures are highly recommended to implement in this certain setting.

## Figures and Tables

**Table 1:** Number and Percentage (%) of *Leptospira* spp. Recovered from the Environmental Samples in the Elephant Camp Areas.

Sampling Locations	Sample Types	*Leptospira*-positive Samples Number (%)				No Growth	Total (%)
		Pathogens Clade		Saprophytes Clade			
		P1	P2	S1	S2		
River	Water	2 ^#^ (25.0%)	3 * (37.5%)	-	1 ^#^ (12.5%)	2 (25.0%)	8 (33.3%)
	Soil	4 * (57.1%)	2 * (28.6%)	-	-	1 (14.3%)	7 (29.2%)
Mud pond	Water	1 * (100%)	-	-	-	0 (0%)	1 (4.2%)
	Soil	-	1 * (50.0%)	1 (50.0%)	-	0 (0%)	2 (8.3%)
Water supplies	Water	-	3 ^#^ (100%)	-	-	0 (0%)	3 (12.5%)
Elephant barn	Soil	-	-	-	-	3 (100%)	3 (12.5%)
Total		7 (29.1%)	9 (37.5%)	1 (4.2%)	1 (4.2%)	6 (25.0%)	24 (100%)
Total *		4 * (16.6%)	5 * (20.8%)	-	-	-	9 * (37.5%)

* The samples were mixed with S1 subclade species based on *secY* PCR and gene sequencing results. # The samples were negative by *secY* PCR.

## Supplementary Materials

The following are available online at https://www.mdpi.com/2414-6366/5/4/183/s1. Table S1: 16S rRNA and *secY* PCR results of the environmental isolates of Leptospira from the elephant camp setting, Table S2: BLASTn analysis results of 16S rRNA and *secY* gene sequences amplified from each environmental isolate.

## References

[1] Levett P.N. (2001). Leptospirosis. Clin. Microbiol. Rev..

[2] Vincent A.T., Schiettekatte O., Goarant C., Neela V.K., Bernet E., Thibeaux R., Ismail N., Khalid M.K.N.M., Amran F., Masuzawa T. (2019). Revisiting the taxonomy and evolution of pathogenicity of the genus *Leptospira* through the prism of genomics. PLoS Negl. Trop. Dis..

[3] Bharti A.R., Nally J.E., Ricaldi J.N., Matthias M.A., Diaz M.M., Lovett M.A., Levett P.N., Gilman R.H., Willig M.R., Gotuzzo E. (2003). Leptospirosis: A zoonotic disease of global importance. Lancet Infect. Dis..

[4] Haake D.A., Levett P.N. (2015). Leptospirosis in Humans. Curr. Top. Microbiol. Immunol..

[5] Goarant C. (2016). Leptospirosis: Risk factors and management challenges in developing countries. Res. Rep. Trop. Med..

[6] Barragán V., Olivas S., Keim P.S., Pearson T. (2017). Critical Knowledge Gaps in Our Understanding of Environmental Cycling and Transmission of *Leptospira* spp.. Appl. Environ. Microbiol..

[7] Gundacker N.D., Rolfe R.J., Rodriguez M. (2017). Infections associated with adventure travel: A systematic review. Travel Med. Infect. Dis..

[8] Hinjoy S., Kongyu S., Doung-Ngern P., Doungchawee G., Colombe S., Tsukayama R., Suwancharoen D. (2019). Environmental and Behavioral Risk Factors for Severe Leptospirosis in Thailand. Trop. Med. Infect. Dis..

[9] Schønning M.H., Phelps M.D., Warnasekara J., Agampodi S.B., Furu P. (2019). A Case–Control Study of Environmental and Occupational Risks of Leptospirosis in Sri Lanka. EcoHealth.

[10] Muñoz-Zanzi C., Groene E., Morawski B.M., Bonner K., Costa F., Bertherat E., Schneider M.C. (2020). A systematic literature review of leptospirosis outbreaks worldwide, 1970–2012. Revista Panamericana de Salud Pública.

[11] Haake D.A., Dundoo M., Cader R., Kubak B.M., Hartskeerl R.A., Sejvar J.J., Ashford D.A. (2002). Leptospirosis, Water Sports, and Chemoprophylaxis. Clin. Infect. Dis..

[12] Koay T.K., Nirmal S., Noitie L., Tan E. (2004). An epidemiological investigation of an outbreak of leptospirosis associated with swimming, Beaufort, Sabah. Med J. Malays..

[13] Guillois Y., Bourhy P., Ayral F., Pivette M., Decors A., Grau J.H.A., Champenois B., Malhère C., Combes B., Richomme C. (2018). An outbreak of leptospirosis among kayakers in Brittany, North-West France, 2016. Eurosurveillance.

[14] Schreiber P.W., Aceto L., Korach R., Marreros N., Ryser-Degiorgis M.-P., Günthard H.F. (2015). Cluster of Leptospirosis Acquired Through River Surfing in Switzerland. Open Forum Infect. Dis..

[15] Casanovas-Massana A., Pedra G.G., Wunder E.A., Diggle P.J., Begon M., Ko A.I. (2018). Quantification of *Leptospira interrogans* Survival in Soil and Water Microcosms. Appl. Environ. Microbiol..

[16] Thibeaux R., Iraola G., Ferrés I., Bierque E., Girault D., Soupé-Gilbert M.-E., Picardeau M., Goarant C. (2018). Deciphering the unexplored Leptospira diversity from soils uncovers genomic evolution to virulence. Microb. Genom..

[17] Saito M., Villanueva S.Y.A.M., Chakraborty A., Miyahara S., Segawa T., Asoh T., Ozuru R., Gloriani N.G., Yanagihara Y., Yoshida S.-I. (2013). Comparative Analysis of *Leptospira* Strains Isolated from Environmental Soil and Water in the Philippines and Japan. Appl. Environ. Microbiol..

[18] Smith D.J.W., Self H.R.M. (1955). Observations on the Survival of *Leptospira australis* A in Soil and Water. Epidemiol. Infect..

[19] Smith C.E.G., Turner L.H. (1961). The effect of pH on the survival of leptospires in water. Bull. World Health Organ..

[20] Thibeaux R., Girault D., Bierque E., Soupé-Gilbert M.-E., Rettinger A., Douyère A., Meyer M., Iraola G., Picardeau M., Goarant C. (2018). Biodiversity of Environmental *Leptospira*: Improving Identification and Revisiting the Diagnosis. Front. Microbiol..

[21] Bierque E., Thibeaux R., Girault D., Soupé-Gilbert M.-E., Goarant C. (2020). A systematic review of *Leptospira* in water and soil environments. PLoS ONE.

[22] Chaiwattanarungruengpaisan S., Suwanpakdee S., Sangkachai N., Chamsai T., Taruyanon K., Thongdee M. (2018). Potentially Pathogenic *Leptospira* Species Isolated from a Waterfall in Thailand. Jpn. J. Infect. Dis..

[23] Boonsilp S., Thaipadungpanit J., Amornchai P., Wuthiekanun V., Chierakul W., Limmathurotsakul D., Day N.P., Peacock S.J. (2011). Molecular detection and speciation of pathogenic *Leptospira* spp. in blood from patients with culture-negative leptospirosis. BMC Infect. Dis..

[24] Thaipadungpanit J., Wuthiekanun V., Chierakul W., Smythe L.D., Petkanchanapong W., Limpaiboon R., Apiwatanaporn A., Slack A.T., Suputtamongkol Y., White N.J. (2007). A Dominant Clone of *Leptospira interrogans* Associated with an Outbreak of Human Leptospirosis in Thailand. PLoS Negl. Trop. Dis..

[25] Gravekamp C., Van De Kemp H., Franzen M., Carrington D., Schoone G.J., Van Eys G.J.J.M., Everard C.O.R., Hartskeerl R.A., Terpstra W.J. (1993). Detection of seven species of pathogenic leptospires by PCR using two sets of primers. J. Gen. Microbiol..

[26] André-Fontaine G., Aviat F., Thorin C. (2015). Waterborne Leptospirosis: Survival and Preservation of the Virulence of Pathogenic *Leptospira* spp. in Fresh Water. Curr. Microbiol..

[27] Stoddard R.A., Bui D., Wuthiekanun V., Haberling D.L., Thaipadungpanit J., Hoffmaster A.R. (2014). Viability of *Leptospira* Isolates from a Human Outbreak in Thailand in Various Water Types, pH, and Temperature Conditions. Am. J. Trop. Med. Hyg..

[28] Parker J., Walker M. (2011). Survival of a pathogenic *Leptospira* serovar in response to combined in vitro pH and temperature stresses. Veter Microbiol..

[29] Hellstrom J., Marshall R. (1978). Survival of *Leptospira interrogans* serovar pomona in an acidic soil under simulated New Zealand field conditions. Res. Veter Sci..

[30] Ali M.R.M., Safiee A.W.M., Yusof N.Y., Fauzi M.H., Yean C.Y., Ismail N. (2018). Isolation of *Leptospira* kmetyi from residential areas of patients with leptospirosis in Kelantan, Malaysia. J. Infect. Public Health.

[31] Masuzawa T., Sakakibara K., Saito M., Hidaka Y., Villanueva S.Y.A.M., Yanagihara Y., Yoshida S. (2017). Characterization of *Leptospira* species isolated from soil collected in Japan. Microbiol. Immunol..

[32] Mendoza M.V., Rivera W.L. (2019). Identification of *Leptospira* spp. from environmental sources in areas with high human leptospirosis incidence in the Philippines. Pathog. Glob. Health.

[33] Narkkul U., Thaipadungpanit J., Srilohasin P., Singkhaimuk P., Thongdee M., Chaiwattanarungruengpaisan S., Krairojananan P., Pan-Ngum W. (2020). Optimization of Culture Protocols to Isolate *Leptospira* spp. from Environmental Water, Field Investigation, and Identification of Factors Associated with the Presence of Leptospira spp. in the Environment. Trop. Med. Infect. Dis..

[34] Scialfa E., Grune S., Brihuega B., Aguirre P., Rivero M. (2018). Isolation of saprophytic Leptospira spp. from a selected environmental water source of Argentina. Revista Argentina de Microbiología.

[35] Pui C.F., Bilung L.M., Apun K., Su’Ut L. (2017). Diversity of *Leptospira* spp. in Rats and Environment from Urban Areas of Sarawak, Malaysia. J. Trop. Med..

[36] Diesch S.L., McCulloch W.F. (1966). Isolation of Pathogenic Leptospires from Waters Used for Recreation. Public Health Rep..

[37] Baker M.F., Baker H.J. (1970). Pathogenic *Leptospira* in Malaysian Surface Waters. Am. J. Trop. Med. Hyg..

[38] Kim J.S. (1987). Leptospirosis: A Newly Identified Disease in Korea. Asia Pac. J. Public Health.

[39] Alexander A.D., Evans L.B., Baker M.F., Baker H.J., Ellison D., Marriapan M. (1975). Pathogenic *Leptospiras* Isolated from Malaysian Surface Waters. Appl. Microbiol..

[40] Lin P.-C., Chi C.-Y., Ho M.-W., Chen C.-M., Ho C.-M., Wang J.-H. (2008). Demographic and clinical features of leptospirosis: Three-year experience in central Taiwan. J. Microbiol. Immunol. Infect..

[41] Sanchez R.G.P., Lopez J.Á., Pereira M.M., Naranjo M.A., Agudelo-Flórez P. (2016). Genetic diversity of *Leptospira* in northwestern Colombia: First report of *Leptospira santarosai* as a recognised leptospirosis agent. Memórias do Instituto Oswaldo Cruz.

[42] Valverde M.D.L.A., Ramirez J., De Oca L.M., Goris M.G., Ahmed N., Hartskeerl R.A. (2008). Arenal, a new *Leptospira* serovar of serogroup Javanica, isolated from a patient in Costa Rica. Infect. Genet. Evol..

[43] Bourhy P., Storck C.H., Theodose R., Olive C., Nicolas M., Hochedez P., Lamaury I., Zinini F., Brémont S., Landier A. (2013). Serovar Diversity of Pathogenic *Leptospira* Circulating in the French West Indies. PLoS Negl. Trop. Dis..

[44] Ganoza C.A., Matthias M.A., Collins-Richards D., Brouwer K.C., Cunningham C.B., Segura E.R., Gilman R.H., Gotuzzo E., Vinetz J. (2006). Determining Risk for Severe Leptospirosis by Molecular Analysis of Environmental Surface Waters for Pathogenic *Leptospira*. PLoS Med..

[45] Miotto B.A., Moreno L.Z., Guilloux A.G.A., De Sousa G.O., Loureiro A.P., Moreno A.M., Lilenbaum W., Vasconcellos S.A., Heinemann M.B., Hagiwara M.K. (2016). Molecular and serological characterization of the first *Leptospira santarosai* strain isolated from a dog. Acta Trop..

[46] Rivera P., Ticlla M., Balda L., Gonzalez D., Céspedes M. (2012). Diversidad genética de aislamientos peruanos de *Leptospira* spp. mediante electroforesis en gel de campo pulsado. Revista Peruana de Medicina Experimental y Salud Pública.

[47] Vasconcellos S.A., Oliveira J.C., Morais Z.M., Baruselli P.S., Amaral R., Pinheiro S.R., Ferreira F., Neto J.S.F., Schönberg A., Hartskeerl R.A. (2001). Isolation of Leptospira santarosai, serovar guaricura from buffaloes (*Bubalus bubalis*) in Vale do Ribeira, São Paulo, Brazil. Braz. J. Microbiol..

[48] Guedes I.B., Araújo S.A.D.A., De Souza G.O., Silva S.O.D.S., Taniwaki S.A., Cortez A., Brandão P.E., Heinemann M.B. (2019). Circulating *Leptospira species* identified in cattle of the Brazilian Amazon. Acta Trop..

[49] Slack A.T., Khairani-Bejo S., Symonds M.L., Dohnt M.F., Galloway R.L., Steigerwalt A.G., Bahaman A.R., Craig S., Harrower B.J., Smythe L.D. (2009). *Leptospira kmetyi* sp. nov., isolated from an environmental source in Malaysia. Int. J. Syst. Evol. Microbiol..

[50] Saito M., Miyahara S., Villanueva S.Y.A.M., Aramaki N., Ikejiri M., Kobayashi Y., Guevarra J.P., Masuzawa T., Gloriani N.G., Yanagihara Y. (2014). PCR and Culture Identification of Pathogenic *Leptospira* spp. from Coastal Soil in Leyte, Philippines, after a Storm Surge during Super Typhoon Haiyan (*Yolanda*). Appl. Environ. Microbiol..

[51] Neela V.K., Azhari N.N., Joseph N., Mimie N.P., Ramli S.N.A., Mustapha N.F., Ishak S.N., Mohd-Taib F.S., Yusof M.A., Desa M.N.M. (2019). An outbreak of leptospirosis among reserve military recruits, Hulu Perdik, Malaysia. Eur. J. Clin. Microbiol. Infect. Dis..

[52] Hochedez P., Escher M., Decoussy H., Pasgrimaud L., Martinez R., Rosine J., Théodose R., Bourhy P., Picardeau M., Olive C. (2013). Outbreak of leptospirosis among canyoning participants, Martinique, 2011. Eurosurveillance.

[53] Athapattu T.P.J., Fernando B.R., Koizumi N., Gamage C.D. (2019). Detection of pathogenic leptospires in the urine of domesticated elephants in Sri Lanka. Acta Trop..

[54] Shivraj D.P., Sanjeevkumar B.M., Sanjukta R., Giridhar P., Renukaprasad C. (2009). Detection of leptospiral antibodies in the sera of captive elephants. Vet. World.

[55] Oni O., Sujit K., Kasemsuwan S., Sakpuaram T., Pfeiffer D.U. (2007). Seroprevalence of leptospirosis in domesticated Asian elephants (*Elephas maximus*) in north and west Thailand in 2004. Veter Rec..

[56] Victoria B., Ahmed A., Zuerner R.L., Ahmed N., Bulach D.M., Quinteiro J., Hartskeerl R.A. (2008). Conservation of the S10-spc-α Locus within Otherwise Highly Plastic Genomes Provides Phylogenetic Insight into the Genus Leptospira. PLoS ONE.

