# Supplementary material for: Potentially Pathogenic Leptospira in the Environment of an Elephant Camp in Thailand
Source: Trop Med Infect Dis. 2020 Dec 6;5(4):183. doi: 10.3390/tropicalmed5040183 (PMC7768412; doi:10.3390/tropicalmed5040183)
Supplement: Supplementary file 1 [file tropicalmed-05-00183-s001.pdf]

Tropical Medicine and Infectious Disease

Table S1. 16S rRNA and *secY* PCR results of the environmental isolates of *Leptospira* from the elephant camp setting.

Isolate	16S rRNA		*secY*
	Pathogenic and intermediate specific primers	Genus specific primers	
EC3	+	ND	+
EC5	-	+	-
EC6	+	ND	+
EC7	+	ND	+
EC8	+	ND	+
EC9	+	ND	+
EC10	-	+	+
EC11	+	ND	+
EC12	+	ND	-
EC13	+	ND	-
EC14	+	ND	+
EC16	+	ND	+
EC17	+	ND	+
EC18	+	ND	+
EC19	+	ND	+
EC20	+	ND	-
EC21	+	ND	-
EC22	+	ND	-
Total	16/18 (89%)	2/2 (100%)	12/18 (67%)

ND: not determined.

Table S2. BLASTn analysis results of 16S rRNA and *secY* gene sequences amplified from each environmental isolate.

Isolate	16S rRNA			*secY*		
	**Probable species**	**E-value**	**Identity (%)**	**Probable species**	**E-value**	**Identity (%)**
EC3	*L. interrogans*	0.0	100	*L. biflexa*	7e-97	88.42
EC5	*L. ognonensis*	0.0	97.75	-	-	-
EC6	*L. kmetyi*	0.0	99.85	*L. biflexa*	7e-97	88.42
EC7	*L. kmetyi*	0.0	100	*L. biflexa*	6e-104	90.18
EC8	*L. johnsonii*<br>*L. koniamboensis*<br>*L. saintgironsiae*<br>*L. neocaledonica*<br>*L. wolffii*	0.0	99.41	*L. biflexa*	6e-104	90.18
EC9	*L. dzianensis*	0.0	97.93	*L. biflexa*	3e-102	89.82
EC10	*L. mtsangambouensis*<br>*L. montravelensis*<br>*L. jelokensis*<br>*L. noumeaensis*<br>*L. bandrabouensis*<br>*L. kemamanensis*<br>*L. ellinghausenii*<br>*L. congkakensis*<br>*L. bouyouniensis*<br>*L. meyeri*<br>*L. yanagawae*<br>*L. levettii*<br>*L. macculloughii*	0.0	100	*L. biflexa*	7e-97	88.42
EC11	*L. johnsonii*<br>*L. koniamboensis*<br>*L. saintgironsiae*<br>*L. neocaledonica*<br>*L. wolffii*	0.0	99.41	*L. biflexa*	7e-97	88.42
EC12	*L. kmetyi*	0.0	99.85	-	-	-
EC13	*L. kmetyi*	0.0	99.85	-	-	-
EC14	*L. dzianensis*	0.0	99.85	*L. santarosai*	1e-125	95.79
EC16	*L. selangorensis*<br>*L. andrefontaineae*<br>*L. haakeii*<br>*L. hartskeerlii*<br>*L. venezuelensis*	0.0	100	*L. santarosai*	3e-50	76.84
EC17	*L. selangorensis*<br>*L. andrefontaineae*<br>*L. haakeii*<br>*L. hartskeerlii*<br>*L. venezuelensis*	0.0	99.41	*L. biflexa*	7e-97	88.42
EC18	*L. johnsonii*<br>*L. koniamboensis*<br>*L. saintgironsiae*<br>*L. neocaledonica*	0.0	99.41	*L. biflexa*	3e-101	89.47
EC19	*L. johnsonii*	0.0	99.41	*L. biflexa*	5e-105	90.53

	L. koniamboensis<br>*L. saintgironsiae*<br>*L. neocaledonica*<br>*L. wolffii*					
EC20	*L. selangorensis*<br>*L. andrefontaineae*<br>*L. haakeii*<br>*L. hartskeerlii*<br>*L. venezuelensis*	0.0	100	-	-	-
EC21	*L. johnsonii*<br>*L. koniamboensis*<br>*L. saintgironsiae*<br>*L. neocaledonica*<br>*L. wolffii*	0.0	99.41	-	-	-
EC22	*L. johnsonii*<br>*L. koniamboensis*<br>*L. saintgironsiae*<br>*L. neocaledonica*<br>*L. wolffii*	0.0	99.41	-	-	-
